# Supplementary material for: IvoryOS: an interoperable web interface for orchestrating Python-based self-driving laboratories
Source: Nat Commun. 2025 Jun 4;16:5182. doi: 10.1038/s41467-025-60514-w (PMC12137583; doi:10.1038/s41467-025-60514-w)
Supplement: Supplementary file 1 — Supplementary Information [file 41467_2025_60514_MOESM1_ESM.pdf]

Supplementary Information

IvoryOS: an interoperable web interface for orchestrating Python-based self-driving laboratories

Wenyu Zhang*[1], Lucy Hao[1], Veronica Lai[2], Ryan Corkery[2], Jacob Jessiman[1], Jiayu Zhang[1], Junliang Liu[2], Yusuke Sato[2], Maria Politi[1], Matthew Reish[1], Rebekah Greenwood[1], Noah Depner[1], Jiyoon Min[1], Rama El-khawaldeh[1], Paloma Prieto[1,2], Ekaterina Trushina[1,3] and Jason E. Hein*[1,2,3,4]

1. Department of Chemistry, The University of British Columbia, Vancouver, BC V6T 1Z1, Canada
2. Telescope Innovations Corp., Vancouver, BC, Canada
3. Department of Chemistry, University of Bergen, Norway
4. Acceleration Consortium, University of Toronto, Toronto, ON, Canada

*Corresponding Author: ivoryzhang@chem.ubc.ca; jhein@chem.ubc.ca

Table of Contents

1 Exported workflow design in JSON 2
2 Complete prompt for the abstract SDL 4
3 Workflow Execution 5
3.1 Workflow script to Python 5
3.2 Closed-loop execution wrapper 5
4 Platform examples 7
4.1 ADC Platform 8
4.2 PurPOSE Platform 10
4.3 Telescope Innovations Solubility Platform 11
4.4 LLE Platform 19
4.5 Telescope Innovations Derivatization Sampling 22
4.6 Flow Chemistry Platform 28
5 SDL serialization 29
5.1 Instance name filter 29
5.2 Complete SDL snapshot 29
6 HTTP request examples 31
6.1 Frontend command example 31
6.2 Backend command execution 31
7 IvoryOS client 32

1 Exported workflow design in JSON

Exported JSON from design in **Fig. 3c**


```json
{
    "name": "workflow_1",
    "deck": "abstract_sdl",
    "status": "editing",
    "script_dict": {
        "cleanup": [],
        "prep": [],
        "script": [
            {
                "action": "dose_solid",
                "arg_types": {
                    "amount_in_mg": "float",
                    "bring_in": "bool"
                },
                "args": {
                    "amount_in_mg": 5.0,
                    "bring_in": true
                },
                "id": 1,
                "instrument": "deck.sdl",
                "return": "mass",
                "uuid": 100653096034927
            },
            {
                "action": "dose_solvent",
                "arg_types": {
                    "amount_in_ml": "float",
                    "name": "str",
                    "rate_ml_per_minute": "float"
                },
                "args": {
                    "amount_in_ml": 5.0,
                    "name": "methanol",
                    "rate_ml_per_minute": 1.0
                },
                "id": 2,
                "instrument": "deck.sdl",
                "return": "",
                "uuid": 36713985591538
            },
            {
                "action": "equilibrate",
```

```
                "arg_types": {
                    "duration": "float",
                    "temp": "float"
                },
                "args": {
                    "duration": 20.0,
                    "temp": 30.0
                },
                "id": 3,
                "instrument": "deck.sdl",
                "return": "",
                "uuid": 208728996131128
            }
        ]
    },
    "time_created": "2024-08-20 14:36:56",
    "last_modified": "2024-09-04 13:30:47",
    "id_order": {
        "cleanup": [],
        "prep": [],
        "script": [
            "1",
            "2",
            "3"
        ]
    },
    "editing_type": "script",
    "author": "admin"
}
```

2 Complete prompt for the abstract SDL

The prompt text green shows the example output JSON format, allowing the generated code to be compatible with the design canvas display in **Fig. 3b**. The prompt text in red is user's question, while the text in blue is the automatically generated instance snapshot, capturing function and parameter features.

```
I have some python functions, for example when calling them I want to write them
using JSON, it is necessary to include all args, for example
def dose_solid(amount_in_mg:float, bring_in:bool=True):
def analyze():

dose_solid(3)
analyze()
I would want to write to
[
{
    "action": "dose_solid",
    "arg_types": {
        "amount_in_mg": "float",
        "bring_in": "bool"
    },
    "args": {
        "amount_in_mg": 3,
        "bring_in": true
    }
},
{
    "action": "analyze",
    "arg_types": {},
    "args": {}
}
]

Now these are my callable functions,
    def analyze():
    def dose_solid(amount_in_mg: float = 5, bring_in: bool = False):
    def dose_solvent(name: str, amount_in_ml: float = 5, rate_ml_per_minute:
float = 1):
    def equilibrate(temp: float, duration: float):

and I want you to find the most appropriate function if I want to do these tasks
"""
dose 10 mg of acetaminophen, and then analyze

"""
and write a list of dictionaries in json accordingly. Please only use these
action names ['analyze', 'dose_solid', 'dose_solvent', 'equilibrate'],
can you also help find the default value you can't find the info from my request.
```

3 Workflow Execution

3.1 Workflow script to Python

Scripted Python functions from design in **Fig. 3c.** This script is prepared locally in the **ivoryos_data/scripts** folder and can also be downloaded using the url link (http://localhost:8000/ivoryos/download/python). Note that preparation and cleanup phases are blank for this design

```python
def workflow_1_cleanup():
    global workflow_1_cleanup

def workflow_1_prep():
    global workflow_1_prep

def workflow_1_script():
    global workflow_1_script
    mass = deck.sdl.dose_solid(**{'amount_in_mg': 5.0, 'bring_in': True})
    deck.sdl.dose_solvent(**{'amount_in_ml': 5.0, 'name': 'methanol',
                             'rate_ml_per_minute': 1.0})
    deck.sdl.equilibrate(**{'duration': 20.0, 'temp': 30.0})
    return {'mass': mass,}
```

3.2 Closed-loop execution wrapper

The configuration format might vary between optimization algorithms. This example closed-loop script runner was built using Ax platform, where the expected parameter and objectives input are as of below code box:

```
parameter=[
     {"name": "param_1", "type": "range", "bounds": [1,2]},
     {"name": "param_1", "type": "range", "bounds": [1,2]}
]
objectives=[
     {"name": "obj_1", "min": True, "threshold": None},
     {"name": "obj_2", "min": True, "threshold": None},
]
```

When submitting the `ivoryOS` Bayesian Optimization option in Fig. 4e, the submitted input would follow the dictionary below:

```
{
    "param_1_type": "range", "param_1_value": [1,2],
    "param_2_type": "range", "param_2_value": [1,2],
    "obj_1_min": True,
    "obj_2_min": True
```

```
    }
```

The gathered configuration input will be converted to the expected format by the `ax_wrapper` function in utils.py

4 Platform examples

Table S1. **Category of SDL Examples**. This table classifies six SDL examples based on their attributes: whether they are robotic arm-based, under construction, externally developed, following flexible automation, or using automation framework.

	Arm-based	Developing	Flexible workflow	External	Framework
**PurPOSE Purification**	Yes	No	Yes	No	No
**ADC**	Yes	No	No	No	No
**LLE**	Yes	Yes	Yes	No	No
**Flow Chem**	No	No	No	No	No
**TI Solubility**	Yes	No	Yes	Yes	Yes
**TI Derivatization Sampling**	No	Yes	Yes	Yes	No

Table S2. Summary of the hardware components four robotic arm-based SDLs that successfully integrated with ivoryOS.

	PurPOSE	ADC	LLE	TI Solubility
**Arm**	Universal Robot UR3e	Kinova Gen 3	Universal Robot UR5e	Universal Robot UR3e
**Temp**	IKA Thermoshaker	IKA Thermoshaker	Mettler Toledo EasyMax 102	IKA magnetic stirrer Stir 250561
**Capping**	Custom capping tool	Custom capping tool	Custom capping tool	TI custom capping tool
**Balance**	Mettler Toledo Quantos	Mettler Toledo Weighing Module	Mettler Toledo Quantos	Mettler Toledo XPR
**Liquid handler**	Sampleomatic + Tecan Cavro pump	Custom mobile liquid handler	Custom mobile liquid handler	Custom mobile liquid handler
**Filtration**	Centrifuge + push filter vial	IKA VACSTAR Vacuum Pump	N/A	N/A
**Analysis**	HPLC	HPLC	HPLC	Computer vision

Table S3. Summary of the hardware components two flow chemistry or sampling-based SDLs that successfully integrated with ivoryOS.

	Flow Chem	TI Derivatization Sampling
**Flow Ractor**	Vapourtec Flow Reactor	N/A
**Analysis**	HPLC	HPLC
**Sampler**	DirectInject™	DirectInject™
**Autosampler**	N/A	Sielc autosampler

4.1 ADC Platform

In this proof-of-concept SDL example,[1] the platform was designed for automated ADC only. The experimental conditions were initially prompted to user in command line. When integrating `ivoryOS` to this platform, the command line prompting method was modified to a function with parameters input (**Figure S1**).

Code:

```
from adc_automation.adc_deck import AdcDeck
# https://gitlab.com/heingroup/adc_automation/-/blob/main/adc_deck.py
import ivoryos

class AdcExample(AdcDeck):
    def initial_conditions_ivoryos(self, desired_dar: float,
                                   ab_molecular_weight: float,
                                   ab_concentration: float,
                                   ab_amount: float,
                                   reduction_concentration: float,
                                   dl_concentration: float,
                                   tcep_concentration: float,
                                   tcep_eq_initial: float):
        """
        Initial conditions: original code used input in command line to prompt
        user for those input. this modified code is based on the original code
        from https://gitlab.com/heingroup/adc_automation/-/blob/main/adc_deck.py
        changing the initial input() to parameter inputs

        :param desired_dar: the desired DAR value
        :param ab_molecular_weight: the antibody molecular weight
        :param ab_concentration: the antibody concentration
        :param ab_amount: the antibody amount
        :param reduction_concentration: the reduction agent concentration
        :param dl_concentration: the drug linker concentration
        :param tcep_concentration: the TCEP concentration
        :param tcep_eq_initial: the initial TCEP equivalence
        """
        self.desired_dar = desired_dar
        self.ab_molecular_weight = ab_molecular_weight
        self.ab_concentration = ab_concentration
        self.ab_amount = ab_amount
        self.reduction_concentration = reduction_concentration
        self.dl_concentration = dl_concentration
        self.tcep_concentration = tcep_concentration
        self.tcep_eq_initial = tcep_eq_initial
        self.initial_reagent_volume_calculations()
```

```
if __name__ == "__main__":

    hplc_dir = r"D:\Chemstation\1\Data\2024-04-25_dar_analysis"
    wavelength_ab = 248
    wavelength_dl = 280

    adc_example = AdcExample(hplc_dir=hplc_dir, wavelength_ab=wavelength_ab,
wavelength_dl=wavelength_dl)

    ivoryos.run(__name__)
```

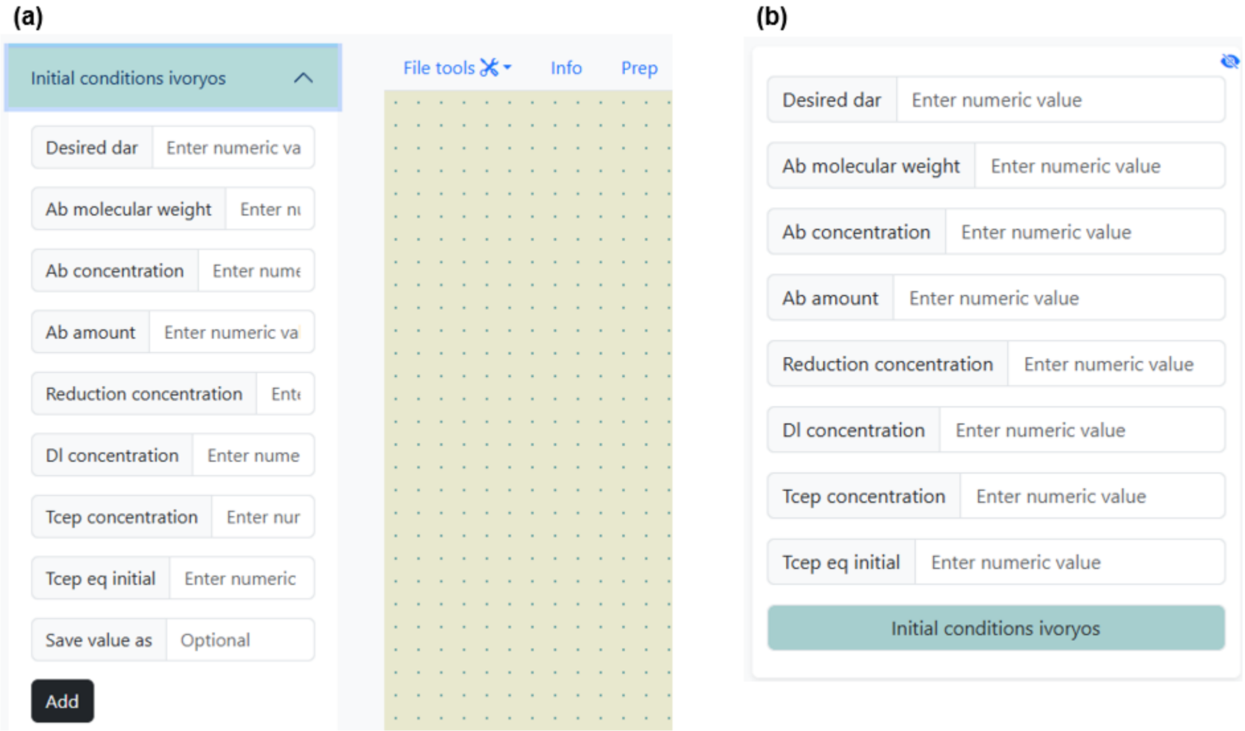

Figure S1. Screenshot of ADC automation parameter input in (a) design interface and (b) control interface.

4.2 PurPOSE Platform

The PurPOSE platform was developed for automated solubility and crystallization screening. In this integration example, instances of hardware connections and workflows are already configured in the script, ur_deck.py and self_driving_config.py respectively. These instances were imported to a new script without modification.

An instruction of the ivoryOS's interoperability, video tutorial of installation and usage, and a demonstration video of handling tasks on PurPOSE Platform: https://youtu.be/dFfJv9I2-1g

code:

```
from ur.self_driving.configuration.self_driving_config import cryst_cosolvent
from ur.configuration.ur_deck import (
     filter_handling,
     vial_handling,
     shaker_handling,
     quantos_handling,
     cap_handling,
     hplc_handling
)

import ivoryos
ivoryos.run(__name__)
```

4.3 Telescope Innovations Solubility Platform

The Telescope solubility platform, developed for automated solubility screening, follows the internal automation framework. To facilitate a more intuitive naming convention and user experience, a wrapper class was created to permit the limited access of only user functions. These user functions are developed with safety checking and a stock database, autonomously choosing the available consumables for execution. The wrapped code for the Telescope Solubility platform:

A demonstration video of the solubility and color matching workflow: https://youtu.be/FC8_dFvfNo4

4.3.1 Solubility workflow

Parameters:

1. `campaign_name`: Campaign name
2. `solid_mass_mg`: Solid mass in mg
3. `solid_name`: Solid sample name
4. `solvent_names`: Solvent name list

Workflow:

1. Load solid dosing head with `solid_name` to automated balance
2. Weigh out an empty vial
3. Open vial cap
4. Add `solid_mass_mg` amount of solid to vial
5. Close vial
6. Add solvent with `solvent_names`
7. Capture with camera
8. Weight out vial

Outputs:

1. No output

Platform Outputs:

1. Experiment data folder
    a. campaign_data.csv
    b. experiment_data.csv
    c. computer vision data

Code:

```
"""
Telescope Innovations Solubility Platform.
Platform Developer Ryan Corkery, Veronica Lai, Telescope Innovations Corp. All
Rights Reserved
"""
from typing import Optional, Dict
import logging

```

```python
import ivoryos

from src.controllers.solubility_controller import SolubilityController
from src import services

logging.getLogger('URRTMonitor').setLevel(logging.INFO)


class SolubilitySDL:
    def __init__(self):
        self.controller = services.services.get_controller(SolubilityController)
        self.controller.wait_for_init()

        self.workflow = self.controller.workflow

    # ------------------------------------------------------------------- #
    # ----------------------------- CONTROLLER --------------------------- #
    # ------------------------------------------------------------------- #

    def run_campaign(self,
                     solid_name: str,
                     solid_mass_mg: float,
                     solvent_names: list,
                     campaign_name: str = None,
                     ):
        solvent_names = ''.join(solvent_names).split(',')
        solvent_names = [s.strip() for s in solvent_names]
        self.controller.run_campaign(solid_name, solid_mass_mg, solvent_names,
campaign_name)

    def run_workflow(self,
                     solid_name: str,
                     solid_mass_mg: float,
                     solvent_name: str,
                     ):
        self.controller.run_workflow(solid_name, solid_mass_mg, solvent_name)

    def set_all_vials_clean(self):
        self.controller.set_all_vials_clean()

    def update_solvent_information(self, name: str, density: float,
valve_position: int):
        self.controller.update_solvent_information(valve_position=valve_position,
name=name, density=density)

```

```
    def get_all_solvent_information(self) -> Dict:
        return self.controller.get_all_solvent_information()

    def update_solid_information(self, name: str, dosing_head_index: str):
        self.controller.update_solid_information(name=name,
index=dosing_head_index)

    def get_all_solid_information(self) -> Dict:
        return self.controller.get_all_solid_information()

    # ------------------------------------------------------------------- #
    # ------------------------ WORKFLOW/SEQUENCES ------------------------ #
    # ------------------------------------------------------------------- #

    def home_liquid_handler(self):
        self.workflow.home_cartesian_gantry()

    def prime_solvent_line(self, line_number: int, volume_ml: float):
        self.controller.prime_solvent_line(line_number=line_number,
volume_ml=volume_ml)

    def start_heating(self, temperature: Optional[float] = None):
        self.workflow.start_heating(temperature=temperature)

    def stop_heating(self):
        self.workflow.stop_heating()

    def start_stirring(self, rpm: Optional[int] = None):
        self.workflow.start_stirring(rpm)

    def stop_stirring(self):
        self.workflow.stop_stirring()

    def auto_recover_arm_position_to_home(self):
        self.controller.auto_recover_arm_position_to_home()



solubility_sdl = SolubilitySDL()

if __name__ == '__main__':
    print('')
    ivoryos.run(__name__, logger=[solubility_sdl.controller.logger.name])
```

Workflow configuration:

Control panel:

campaign_name	solid_mass_mg	solid_name	solvent_names
ivory_demo	5	NAPROXEN	ACETONITRILE, I
ivory_demo	5	ISO	ACETONITRILE, I
ivory_demo	5	NAPROXEN	METHANOL

Figure S2. Screenshot of solubility workflow configuration parameters

Workflow results:

Table S4. Results of 3 iterations of Telescope Solubility workflow.

IvoryOS iteration	id	campaign_name	experiment_number	vial_index	solid	solvent
**Iteration 1**	1	ivory_demo_2025-01-07_T135632	1	A2	NAPROXEN	ACETONITRILE
	2	ivory_demo_2025-01-07_T135632	2	A3	NAPROXEN	METHANOL
**Iteration 2**	1	ivory_demo_2025-01-07_T142411	1	A4	ISO	ACETONITRILE
	2	ivory_demo_2025-01-07_T142411	2	A5	ISO	METHANOL
**Iteration 3**	1	ivory_demo_2025-01-07_T145136	1	A6	NAPROXEN	METHANOL

Table S2 cont.

final_state	solubility_mg_per_ml	solvent_volume_ul	actual_solvent_volume_ul	solvent_percent_error	solid_mass_mg
dissolved	0.783	1600	-1155.8621	238.424821	5
dissolved	0.791	1600	-1084.1969	247.574671	5
dissolved	0.783	1600	-1125.4023	242.1713818813	5
dissolved	0.791	1600	-1097.8959	245.7333110795	5
dissolved	0.791	1600	-1072.5262	249.1805017144	5

Table S2 cont.

actual_solid_mass_mg	solid_percent_error	start_time	completed_time	empty_capped_vial_mass_mg	empty_uncapped_vial_mass_mg
4.955	0.90817356	56:32.4	10:26.0	2428.07	2148.34
5.26	4.94296578	10:26.0	23:57.4	2379.44	2095.135
5.135	2.62901655306	24:11.2	37:59.4	2406.9	2123.935
5.135	2.62901655306	37:59.4	51:22.6	2382.705	2105.42
5.39	7.23562152133	51:36.8	05:24.2	2371.235	2094.545

Table S2 cont.

data_folder
C:\git_repositories\solubility\data\ivory_demo_2025-01-07_T135632\experiment_1
C:\git_repositories\solubility\data\ivory_demo_2025-01-07_T135632\experiment_2
C:\git_repositories\solubility\data\ivory_demo_2025-01-07_T142411\experiment_1
C:\git_repositories\solubility\data\ivory_demo_2025-01-07_T142411\experiment_2
C:\git_repositories\solubility\data\ivory_demo_2025-01-07_T145136\experiment_1

4.3.2 Color matching optimization

The same platform was modified to execute an autonomous color matching experiment. Dilutions of red and blue food coloring were prepared to mix to a target color.

Parameter:

1. `solvent_1_ul`: blue dye volume in µL

Workflow:

1. Move empty vessel to the stir station, and start stirring
2. Add `solvent_1_ul` volume of blue dye
3. Add 1500 – `solvent_1_ul` volume of red dye
4. Take a vessel capture
5. Move vessel back to the tray

Output:

1. `score`: difference score to target color

Code:

```
from typing import Optional, Dict
import logging

import ivoryos

from color_matching import ColorAnalyzer
from src.controllers.colour_matching_controller import ColourMatchingController
from src import services


logging.getLogger('URRTMonitor').setLevel(logging.INFO)
_reference_image_path =
r"C:\git_repositories\solubility\data\ivory_colour_matching\blue 1000ul red
500ul.png"
_roi_coords = (1150, 650, 400, 400)  # Adjust these coordinates to match your ROI

class ColourMatchingSDL:
    def __init__(self):
        self.controller =
services.services.get_controller(ColourMatchingController)
        self.controller.wait_for_init()

        self.analyzer = ColorAnalyzer(_reference_image_path, _roi_coords)
        self.workflow = self.controller.workflow

    # --------------------------------------------------------------------- #
    # ----------------------------- CONTROLLER --------------------------- #
    # --------------------------------------------------------------------- #
    # -------------------------------- ... ------------------------------- #
```

```python
    # ------------------------- full code in gitlab ---------------------- #
    # -------------------------------- ... ------------------------------- #

    def create_vial_mixture(self, solvent_1_ul: int):
        solvent_2_ul = 1500 - solvent_1_ul
        frame = self.controller.create_vial_mixture(solvent_1_ul=solvent_1_ul,
solvent_2_ul=solvent_2_ul)
        return self.analyzer.analyze_image(frame)


colour_matching_sdl = ColourMatchingSDL()


if __name__ == '__main__':
    print('')
    ivoryos.run(__name__, logger=[colour_matching_sdl.controller.logger.name])
```

Optimization configuration:
The solvent volume range was set to [1, 1500]. The objective was set to minimize the difference score function to the target color in lab color space. The iteration was set to 20 for proof-of-concept demonstration.

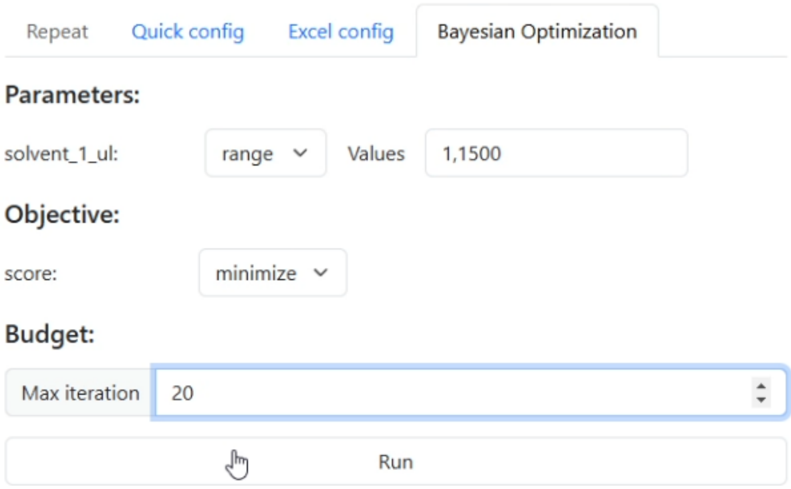

Figure S3. Screenshot of Bayesian Optimization configuration parameters.

Optimization results:

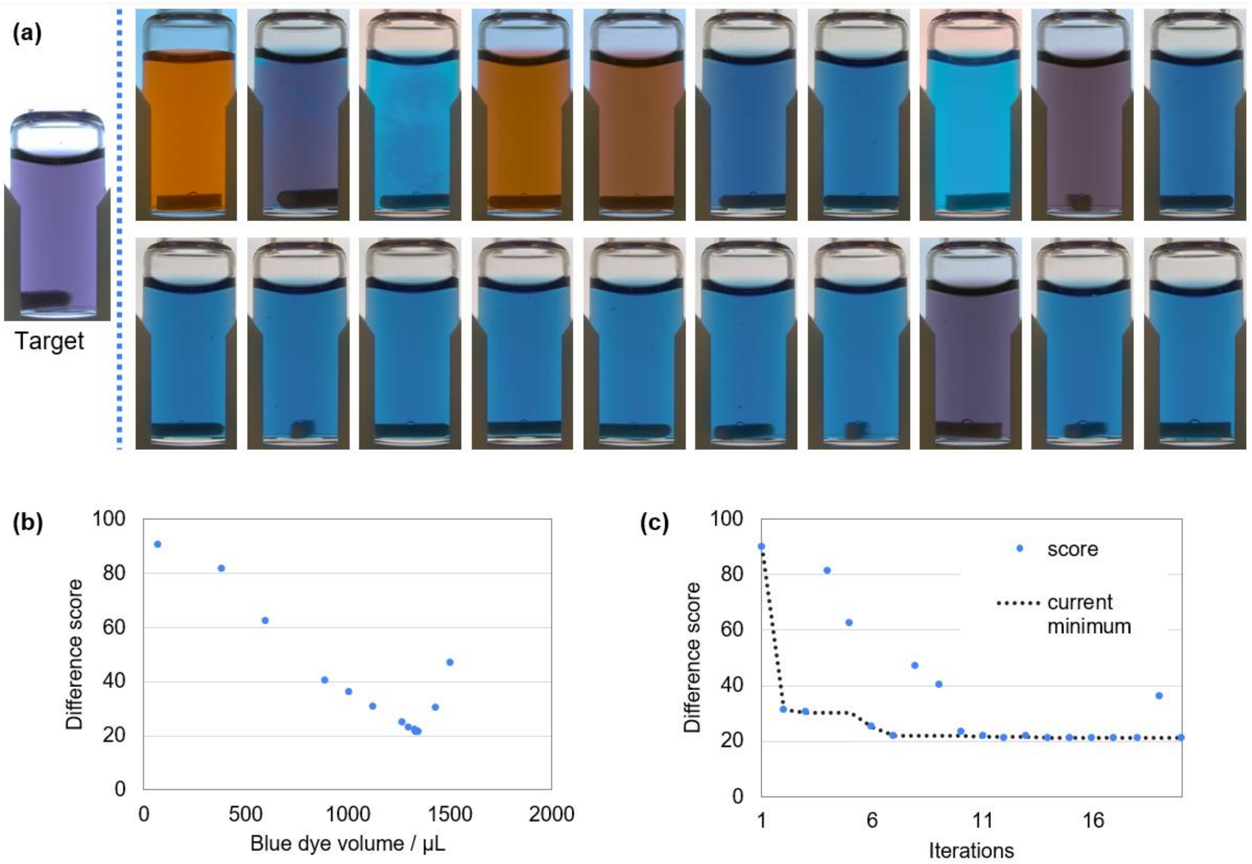

Figure S4. Optimization result of color matching experiments. a. Images of vials showing the target color and progression toward the target color across 20 iterations. **b.** Difference score as a function of blue dye volume. **c.** Iterative optimization process (dotted line: current minimum difference score).

4.4 LLE Platform

In this example, user can design collaborative actions between robotic arm, mobile liquid handlers and balance. For example, weighing vial located on the vial tray using arm and balance (**Figure S5**). User can download the Python script (**Figure S6**) and modify the code according to their function use cases.

Code:

```
from automated_lle.implementations.SDL7.sdl7_ur_manager import ArmManager
from automated_lle.implementations.SDL7.sdl7_mlh import MLH
from automated_lle.components.balance_control import Quan
from automated_lle.components.lc_control import HPLC
from automated_lle.components.logger import logger, file_log
import ivoryos

file_log(enable=True, path="D:/git_repositories/automated-
lle/implementation/SDL7/logs")
logger.info("Redo from 2025-01-27")
logger.info("Running Navy Blue Dye extraction example with wash")

# import your sequence(s) from a URScript file
sequence = r"D:\git_repositories\automated-
lle\automated_lle\components\sequences\LLE_Deck.script"
# create an instance o UR3Arm
ur_address = "137.82.65.187"
ur = ArmManager(ur_address, sequence)
mlh = MLH(mlh_port='COM3', rinse_port='COM4')
balance= Quan()
hplc= HPLC()

if __name__ == "__main__":
    ivoryos.run(__name__)
```

Parameters:

1. `slot`: vial location on the tray

Workflow:

1. Robotic arm picks up HPLC vial from `slot`
2. Robotic arm rotates to vertical safe location
3. Zero the balance
4. Robotic arm places HPLC vial to the balance
5. Balance closes the side door
6. Robotic arm picks up HPLC vial from the balance
7. Robotic arm rotates to horizontal safe location
8. Robotic arm returns HPLC vial to `slot`

Outputs:

1. Weight

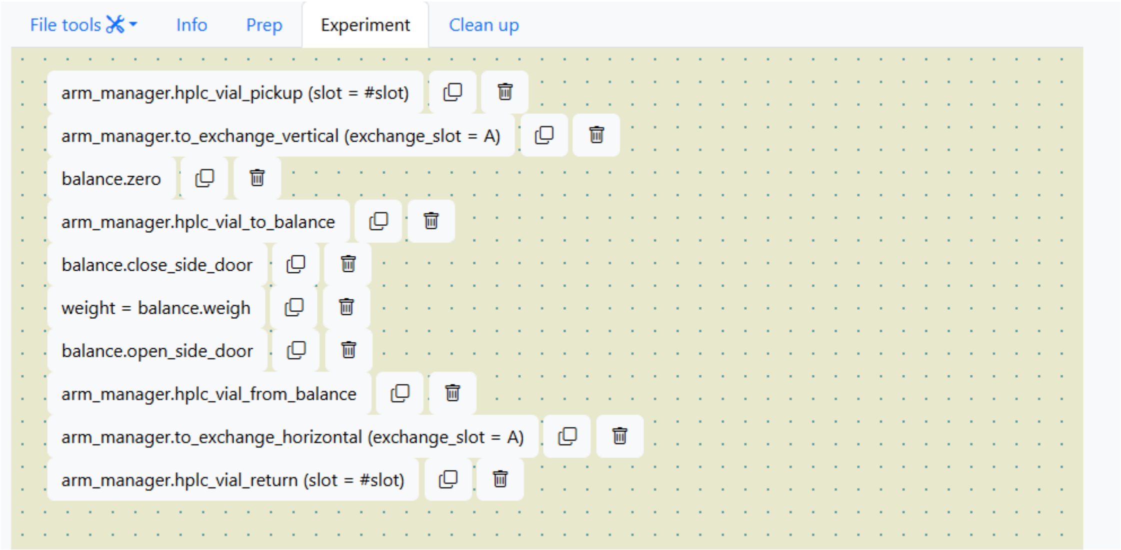

Figure S5. Screenshot of a task design performing vial weighing built from balance module and arm manager using ivoryOS's workflow design.

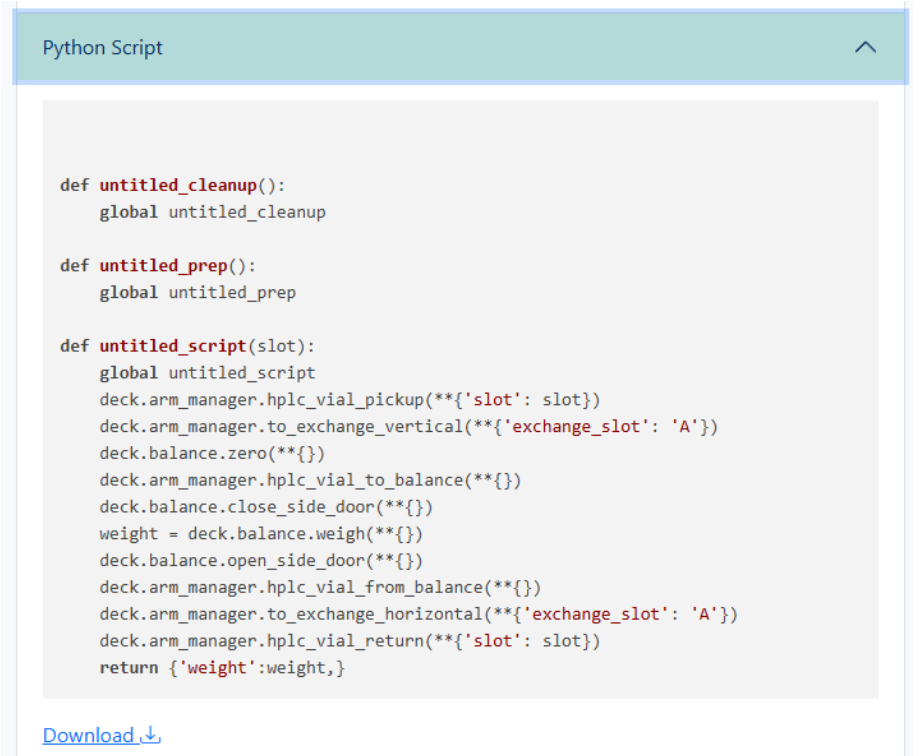


```
def untitled_cleanup():
    global untitled_cleanup

def untitled_prep():
    global untitled_prep

def untitled_script(slot):
    global untitled_script
    deck.arm_manager.hplc_vial_pickup(**{'slot': slot})
    deck.arm_manager.to_exchange_vertical(**{'exchange_slot': 'A'})
    deck.balance.zero(**{})
    deck.arm_manager.hplc_vial_to_balance(**{})
    deck.balance.close_side_door(**{})
    weight = deck.balance.weigh(**{})
    deck.balance.open_side_door(**{})
    deck.arm_manager.hplc_vial_from_balance(**{})
    deck.arm_manager.to_exchange_horizontal(**{'exchange_slot': 'A'})
    deck.arm_manager.hplc_vial_return(**{'slot': slot})
    return {'weight':weight,}
```

Figure S6. Screenshot of generated Python script for performing vial weighing built from balance module and arm manager using ivoryOS's workflow design.

Cleaned code from **Figure S6:**

```
def untitled_script(slot):
    arm_manager.hplc_vial_pickup(**{'slot': slot})
    arm_manager.to_exchange_vertical(**{'exchange_slot': 'A'})
    balance.zero(**{})
    arm_manager.hplc_vial_to_balance(**{})
    balance.close_side_door(**{})
    weight = balance.weigh(**{})
    balance.open_side_door(**{})
    arm_manager.hplc_vial_from_balance(**{})
    arm_manager.to_exchange_horizontal(**{'exchange_slot': 'A'})
    arm_manager.hplc_vial_return(**{'slot': slot})
    return {'weight':weight,}
```

4.5 Telescope Innovations Derivatization Sampling

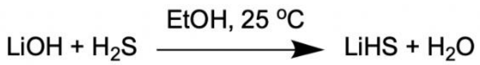

This platform was developed to monitor the oxygen containing and S-containing Lithium compound in the above reaction. The derivatization solution is Benzoyl chloride + pyridine + acetonitrile solution (2:4:10 by volume). Derivatization solution will react with water or LiOH to form benzoic anhydride, and will react with LiHS or Li2S to form benzoic thioanhydride, pyridine is a nucleophilic catalyst, which accelerate the reaction. NaOMe solution will quench the excess Benzoyl chloride to methyl benzoate. NaOMe will also react with benzoic anhydride and benzoic thioanhydride to form benzoic acid and benzothioic (S-acid). The change of oxygen containing compound (LiOH or water) and the S-containing compound (LiHS) in the reaction is monitored by monitoring the peak area of benzoic acid (O-acid) and benzothioic (S-acid) using HPLC (**Figure S7**).

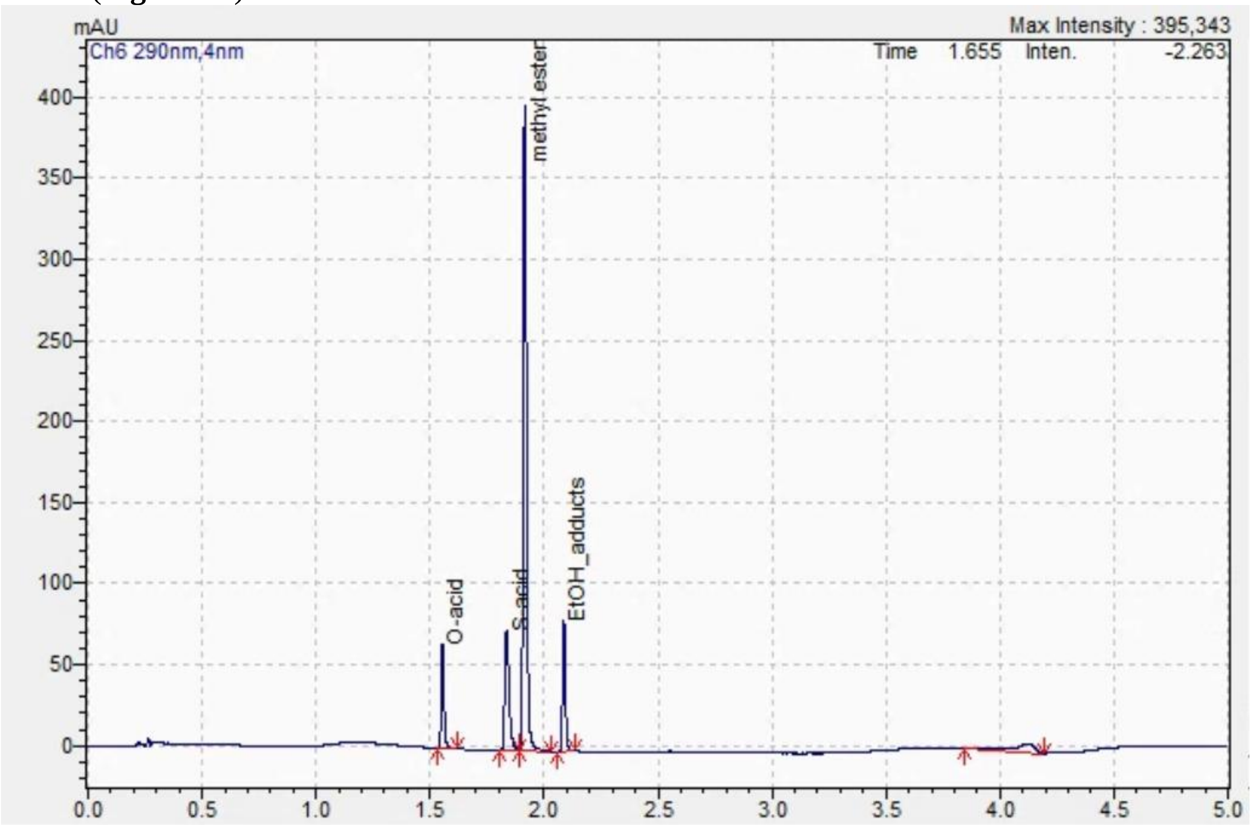

Figure S7. Chromatograph of benzoic acid and benzothioic (S-acid) for monitoring the change of oxygen containing compound (LiOH or water) and the S-containing compound (LiHS) in the reaction.

Parameters:

1. `vial_number`: vial number on Sielc automation

Workflow:

1. Sampler extends, use derivatization solution (port 4) to purge line
2. Sampler retracts, the sample aliquot gets react with the derivatization solution

3. Push with 0.9 mL of any solvent
4. Push 0.1 mL of the derivatized sample into vial with `vial_number`
5. Prime the line with port 1 which is 50 mM NaOMe solution in MeOH
6. Push 0.9 mL of port 1 solution into the same `vial_number`
7. Wash the line with EtOH, which is reaction solvent, to prepare for next sampling

Output:

1. No output

Code:

```
import os
from vicivalve import VICI
from ftdi_serial import Serial
from sielc_dompser.autosampler.autosampler import Autosampler

class SimpleAutosampler:
    def __init__(self):
        self.autosampler = Autosampler('COM12')

    def home_autosampler(self):
        self.autosampler.home_tray_arm()

    def move_needle_to_injection_port(self):
        self.autosampler.needle_to_bottom_of_injection_port()

    def move_needle_to_waste_port(self):
        self.autosampler.needle_to_bottom_of_waste_port()

    def move_needle_to_bottom_of_vial(self, vial_number: int):
        self.autosampler.needle_to_bottom_of_vial(vial_number)



# todo set this
os.environ['DI_HOST'] = '192.168.254.199'  # '192.168.2.98' # kepler
os.environ['LABSOLUTIONS_BASE_ADDRESS'] = 'http://localhost:8000'

from directinject import *


class CustomSequence:
    def __init__(self):
        pass

    def sample_with_sielc(self, push_to_waste: float, vial_number: int):
        sielc.move_needle_to_waste_port(**{})
        sampler.extend(**{})
```

```python
        source_valve.set_position(**{'position': 4})
        delivery_pump.dispense(**{'flow_rate': '4', 'volume': '2', 'wait': True})
        sampler.retract(**{})
        source_valve.set_position(**{'position': 1})
        delivery_pump.dispense(**{'flow_rate': '4', 'volume': push_to_waste, 'wait': True})
        sielc.move_needle_to_bottom_of_vial(**{'vial_number': vial_number})
        delivery_pump.dispense(**{'flow_rate': 0.5, 'volume': 0.1, 'wait': 'True'})
        sielc.move_needle_to_waste_port(**{})
        delivery_pump.dispense(**{'flow_rate': '4', 'volume': '2', 'wait': True})
        sielc.move_needle_to_bottom_of_vial(**{'vial_number': vial_number})
        delivery_pump.dispense(**{'flow_rate': 0.5, 'volume': 0.9, 'wait': 'True'})
        sielc.move_needle_to_waste_port(**{})
        source_valve.set_position(**{'position': '2'})
        delivery_pump.dispense(**{'flow_rate': '4', 'volume': '4', 'wait': True})


# _vici_serial = Serial(device_serial='DT04576V', baudrate=9600)
#
# vici_valve = VICI(_vici_serial)

sielc = SimpleAutosampler()
custom_sequence = CustomSequence()



import ivoryos

ivoryos.run(__name__, port=8001)
```

Design:

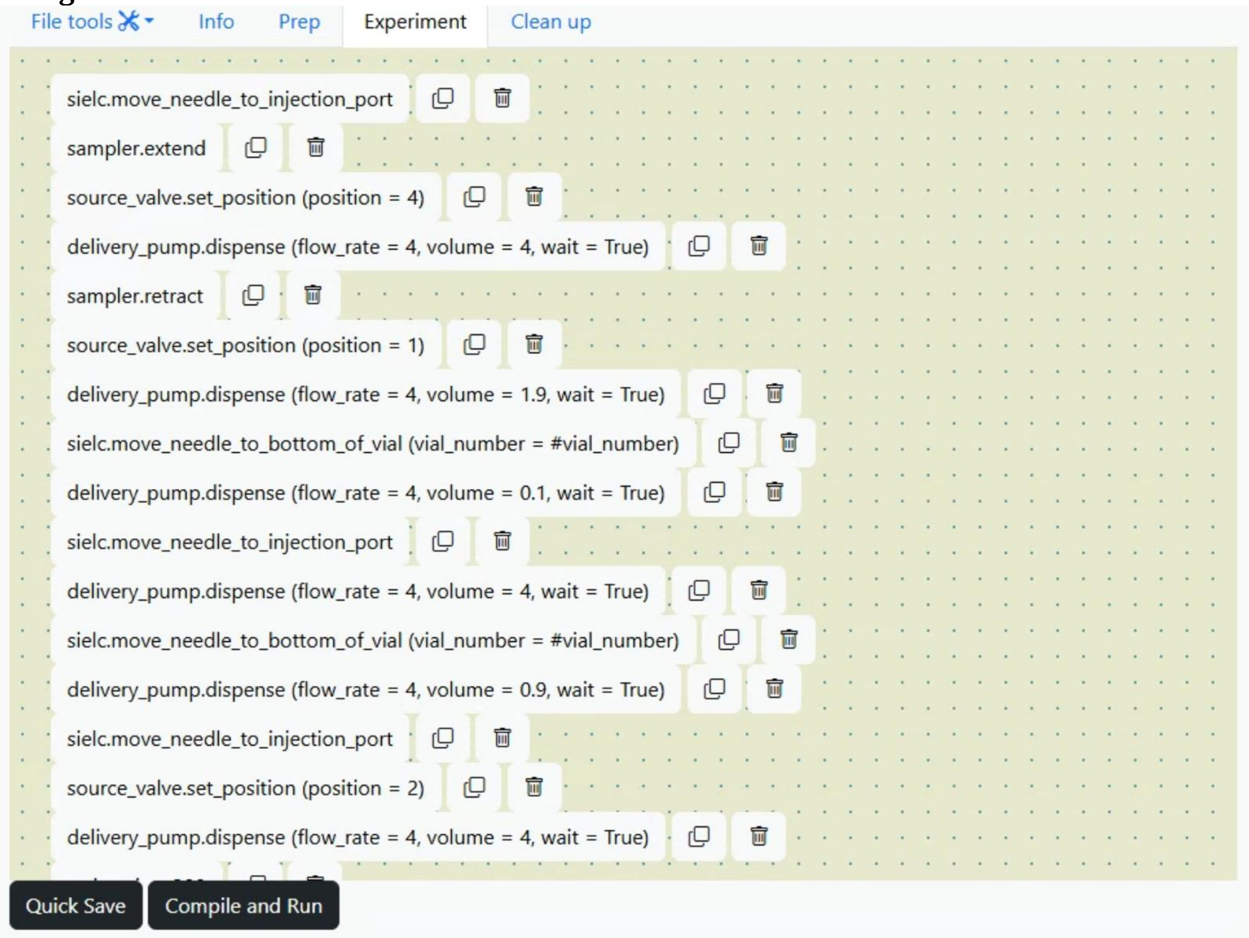

Figure S8. Screenshot of workflow design using `ivoryOS`.

Configuration:

The vial number was configured using the Excel configuration option and set to [1, 2, 3, 4, 5, 6, …, 34] to sequentially use the location on the Sielc autosampler.

Table S5. Vial number configuration and results of 34 iterations of Telescope Derivatization sampling workflow.

**Configuration**	**HPLC data**		
**vial_number**			
**int**	Time (h)	O-acid	S-acid
**1**	0	44,129	0
**2**	0.0775008	42,871	0
**3**	0.155556	46,491	0
**4**	0.4755576	51,437	0
**5**	0.5544456	48,977	0
**6**	0.7600008	47,274	0
**7**	0.838056	51,289	0
**8**	0.9338904	61,601	0
**9**	1.0113888	51,417	0
**10**	1.089168	52,801	10032
**11**	1.2102792	53,952	24342
**12**	1.2888888	54,836	33653
**13**	1.3675008	53,558	43205
**14**	1.4463888	55,474	52182
**15**	1.5247224	51,476	67692
**16**	1.623612	55,831	85914
**17**	1.7016672	54,504	97484
**18**	1.7797224	61,852	111048
**19**	1.8575016	53,972	139629
**20**	1.9358352	60,887	166922
**21**	2.0147232	57,814	196261
**22**	2.0933352	59,207	226078
**23**	2.16	62,020	256896
**24**	2.25	63,041	287401
**25**	2.3283336	70,013	373153
**26**	2.4063888	64,716	350507
**27**	2.4850008	68,371	417115
**28**	2.563056	69,773	420226
**29**	2.641668	68,621	420112
**30**	2.7200016	69,944	432927
**31**	2.799168	71,311	439833
**32**	3.243612	69,151	425191
**33**	3.3211128	71,174	428882
**34**	3.399168	69,959	419707

Workflow results:

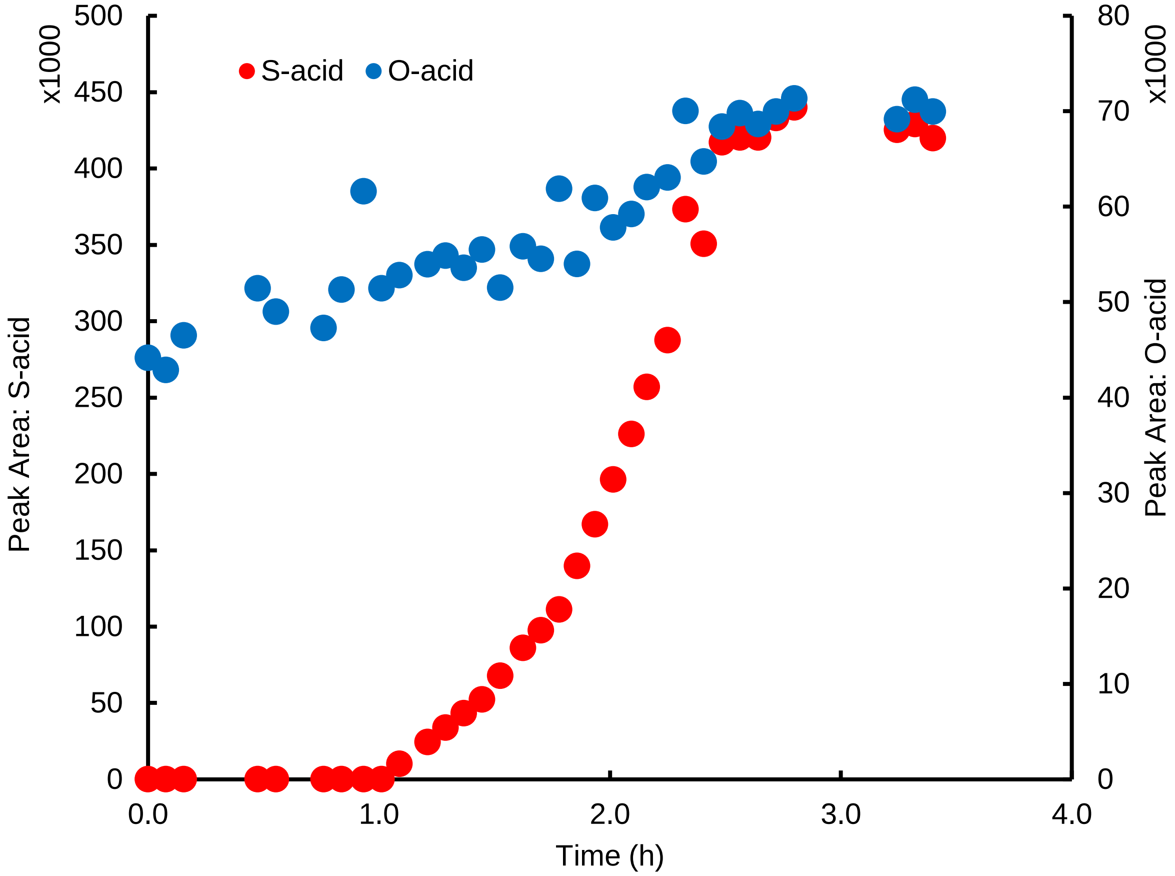

Figure S9. Online HPLC monitoring of benzoic acid (O-acid) and benzothioic (S-acid) for monitoring the change of oxygen containing compound (LiOH or water) and the S-containing compound (LiHS) in the reaction.

4.6 Flow Chemistry Platform

Parameters:

2. ester_eq: ester equivalence
3. ammonia_eq: ammonia equivalence
4. residence_time: residence time
5. temperature: temperature

Workflow:

1. calculate flowrate according to residence time and reagents equivalence
2. start pumps and tempering on flow chemistry reactor

Output:

2. score function of product peak area and parameters

Code:

```
from flow_optimizer import FlowOptimizer
import ivoryos

if __name__ == "__main__":
    flow_optimizer = FlowOptimizer()

    ivoryos.run(__name__, logger="flow")
```

Optimization configuration:

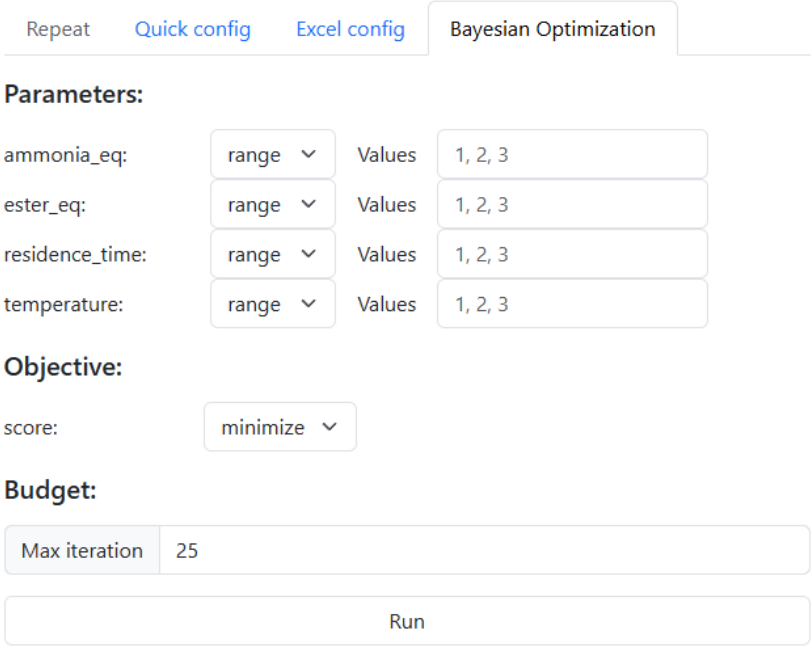

Figure S10. Screenshot of Bayesian Optimization configuration parameters.

5 SDL serialization

5.1 Instance name filter

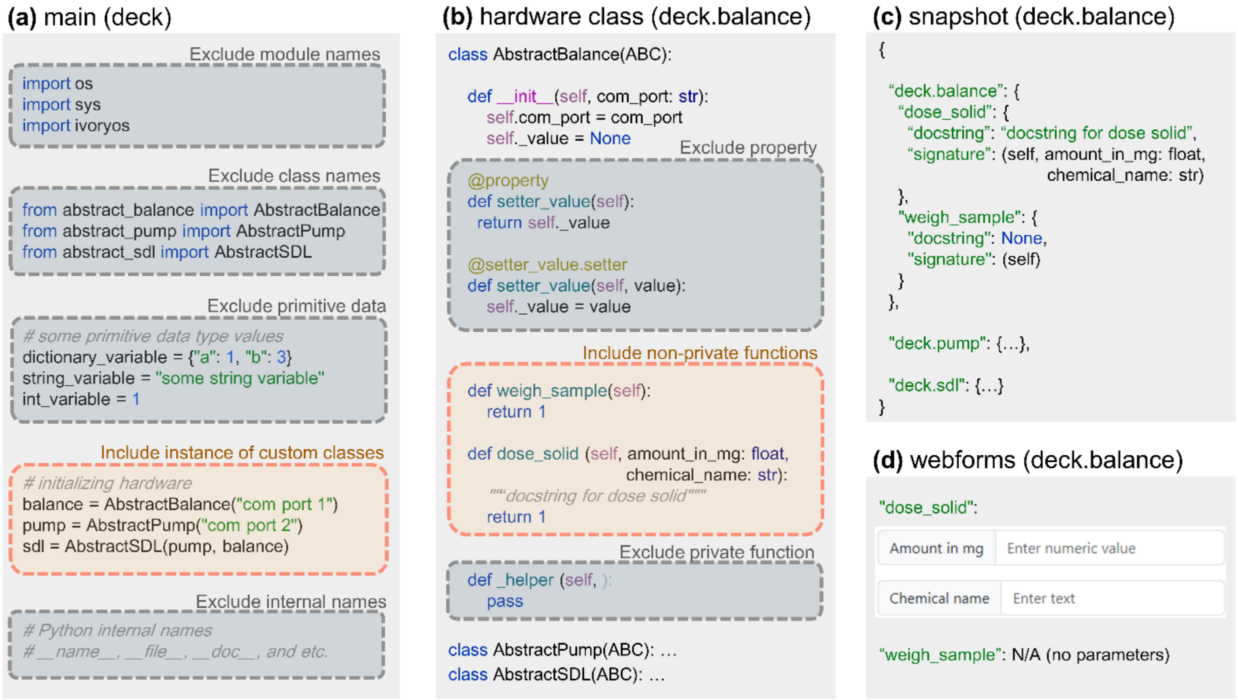

Figure S11. Instance filter rules in SDL serialization. a Main source SDL script in abstract_sdl_example/abstract_sdl.py. Only instances of custom classes that are highlighted in orange are serialized. **b** An example of module class (deck.balance) where only non-private functions are extracted during serialization. **c** Snapshot of the SDL control script with expanded deck.balance dictionary. **d** Rendered web forms with tailored parameter entries.

Code:

```
snapshot = {f"deck.{name}": _inspect_class(val) for name, val in vars(deck).items()
            if not type(val).__module__ == 'builtins'
            and not name[0].isupper()
            and not name.startswith("_")}
```

5.2 Complete SDL snapshot

Python dictionary generated for the abstract SDL example in **Fig. S11**.

```
{
    'deck.balance': {
        'dose_solid': {
            'signature': <Signature (self, amount_in_mg: float)>,
            'docstring': 'this function is used to dose solid'
        },
```

```
        'weigh_sample': {
            'signature': <Signature (self)>,
            'docstring': None
        }
    },
    'deck.pump': {
        'dose_liquid': {
            'signature': <Signature (self, amount_in_ml: float, rate_ml_per_minute: float)>,
            'docstring': None
        }
    },
    'deck.sdl': {
        'analyze': {
            'signature': <Signature (self, param_1: int, param_2: int)>,
            'docstring': None
        },
        'dose_solid': {
            'signature': <Signature (self, amount_in_mg: float = 5, bring_in: bool = False)>,
            'docstring': 'dose current chemical'
        },
        'dose_solvent': {
            'signature': <Signature (self, name: str, amount_in_ml: float = 5, rate_ml_per_minute: float = 1)>,
            'docstring': None
        },
        'equilibrate': {
            'signature': <Signature (self, temp: float, duration: float)>,
            'docstring': None
        },
    },
    'deck_name': 'abstract_sdl'
}
```

6 HTTP request examples

6.1 Frontend command example

To call method for balance instance in the abstract SDL example

```
balance.dose_solid(amount_in_mg=10, chemical_name="solid_1")
```

through HTTP using Python `requests` when `ivoryOS` is launched

```
import requests

session = requests.Session()
response = session.post(url='http://localhost:8000/backend_control/deck.balance',
                        data={
                            "hidden_name": "dose_solid",
                            "amount_in_mg": 10,
                            "chemical_name": "solid_1"
                        })
```

6.2 Backend command execution

`find_instrument_by_name` is used to find instance that loaded in `ivoryOS` global configuration

```
@control.route("/backend_control/<instrument>", methods=['POST'])
def backend_control(instrument):
    instance = find_instrument_by_name(instrument) # balance
    kwargs = request.form.copy()
    method_name = kwargs.pop("hidden_name")
    method_executable = getattr(instance, method_name)
    output = method_executable(**{"amount_in_mg": 10,
                                  "chemical_name": "solid_1"})
    return output
```

7 IvoryOS client

getting snapshot from the server:

```
snapshot = session.get("http://localhost:8000/backend_client").json()
```

using `client.generate_proxy_script(url)` to generate the Balance API for `AbstractBalance`

```
class Balance:
    url = "http://localhost:8000/backend_control/deck.balance"
    def dose_solid(self, amount_in_mg: float, chemical_name: str):
        """this function is used to dose solid"""
        session.post(self.url, data={"hidden_name": "dose_solid",
                                     "amount_in_mg": amount_in_mg,
                                     "chemical_name": chemical_name})

    def weigh_sample(self):
        session.post(self.url, data={"hidden_name": "weigh_sample"})
```
